# The genus *Brulleia* Szépligeti (Hymenoptera, Braconidae, Helconinae) from China, with descriptions of four new species

**DOI:** 10.3897/zookeys.257.3832

**Published:** 2013-01-04

**Authors:** Cheng-jin Yan, Jun-hua He, Xue-xin Chen

**Affiliations:** 1 State Key Laboratory of Rice Biology and Ministry of Agriculture Key Lab of Agricultural Entomology, Institute of Insect Sciences, Zhejiang University, Hangzhou 310058, China

**Keywords:** Hymenoptera, Braconidae, Helconinae, *Brulleia*, new species, China

## Abstract

The species of the genus *Brulleia* Szépligeti, 1904 (Hymenoptera, Braconidae, Helconinae) from China are revised. Four new species, namely *Brulleia fanjingensis* YanandChen, **sp. n.**, *Brulleia longipalpis* YanandChen, **sp. n.**, *Brulleia noncarinata* YanandChen, **sp. n.** and*Brulleia punctata* Yan andChen, **sp. n.** are described and illustrated. A key to the Chinese species of the genus *Brulleia* is included.

## Introduction

The genus *Brulleia* Szépligeti, 1904 (Hymenoptera, Braconidae, Helconinae, Brulleiini) contains 18 valid species and is distributed in the eastern Palaearctic, Oriental and Australasian regions (van Achterberg 1983, 1993; Chen et al. 1993; Chou and Hsu 1998). The biology of this genus is largely unknown, but one species, *Brulleia obereae* Chen and van Achterberg, 1993 is reported as parasitoid of larvae of *Oberea* sp. (Coleoptera, Cerambycidae) (Chen et al., 1993).

Ten species were already recorded from China (Chen et al. 1993; Chen et al. 1998, 2001; Chou and Hsu 1998). In the present paper additional four new species of this genus are described and illustrated from Guizhou, Tibet and Hebei, the western and northern parts of China: *Brulleia fanjingensis* YanandChen, sp. n., *Brulleia longipalpis* YanandChen, sp. n., *Brulleia noncarinata* YanandChen, sp. n., and *Brulleia punctata* Yan and Chen sp. n.They are described and illustrated in detail, and a key to all Chinese species of *Brulleia* is updated.

## Material and methods

The terminology and measurements used follow van Achterberg (1983, 1988, 1993) and Chen et al. (1993). Additional sources for the description of sculpture and setation are Belokobylskij (1998). The following abbreviations are used for morphology: POL – postocellar line; OOL – ocular-ocellar line; OD – maximum diameter of lateral ocellus. Type specimens and other materials are deposited in the Parasitic Hymenoptera Collection of Zhejiang University, Hangzhou, China (ZJUH) and Shanghai Entomological Museum, Chinese Academy of Sciences, Shanghai, China (SEMS), respectively.

Descriptions and measurements were made under a stereomicroscope (Zeiss Stemi SV 6). All figures were made with a Leica DFC425 Camera attached to a stereomicroscope (Leica M205 A, Germany) and Leica Application Suite version 3.60 software.

## Taxonomy

### Brulleia

Szépligeti, 1904

http://species-id.net/wiki/Brulleia

Brulleia Szépligeti, 1904: 150; Shenefelt 1970: 190; van Achterberg 1983: 287; Chen et al. 1993: 378; Chou and Hsu 1998: 284.

#### Type species.

*Brulleia melanocephala* Szépligeti, 1904.

#### Diagnosis.

Mandibles evenly curved; maxillary and labial palpi with 2–6 and 2–3 segments, respectively; face densely reticulate-rugose; clypeus more or less convex or medially depressed; occipital carina arched medio-dorsally or reduced; vertex usually with longitudinal groove; frons weakly concave medially or nearly flat; length of hind tibia 1.6–2.0 times hind femur; second tergite smooth or sculptured basally; vein 1-SR of fore wing absent; vein 2A of hind wing absent.

#### Host.

Larvae of *Oberea* sp. (Cerambycidae).

#### Distribution.

China (Hebei, Zhejiang, Jiangxi, Sichuan, Fujian, Guangxi, Yunnan, Guizhou, Tibet); East Palaearctic, Oriental and Australasian regions.

### Brulleia fanjingensis

Yan &Chen, sp. n.

urn:lsid:zoobank.org:act:C1FEE184-259D-4DB6-92BB-2779E2CF7366

http://species-id.net/wiki/Brulleia_fanjingensis

Figs 1–10

#### Material examined.

Holotype, ♂, China, Guizhou Prov., Fanjing Mountain Gokokuji Temple, 1000 m, 4.VIII.2001, Ma Yun, No. 200108606 (ZJUH).

#### Description.

Body length 15.5 mm. Fore wing length 10.0 mm.

Head. Antennal segments 42; third segment 1.7 times longer than fourth segment; length of third, fourth and penultimate segments 5.3, 4.3 and 2.0 times their width, respectively. Maxillary palp 4-segmented; labial palp 3-segmented; length of maxillary palp 0.6 times height of head. Head in dorsal view 0.6 times as long as wide. Eye 1.4 times as long as temple in dorsal view. Length of malar space 0.7 times basal width of mandible, 0.4 times maximum width of eye. POL: OD: OOL=10: 8: 40. Temple densely punctate dorsally, coarsely rugose ventrally. Vertex densely punctate. Frons weakly concave, medially with coarsely transverse rugae, laterally with coarsely oblique striae. Face reticulate-punctate. Clypeus reticulate-punctate dorsally, with median notch on upper margin. Mandibles striated at basal 0.6. Labium with its apical margin convex medially.

Mesosoma. Almost twice as long as its height. Pronope spindle-shaped. Side of pronotum punctate, antero-medially and subdorsally crenulate, ventro-posteriorly rugose-punctate. Mesoscutum punctate, middle lobe with weak longitudinal groove medially. Notauli narrow and deep, crenulate, its posterior area with median carina. Scutellum rather flat and densely punctate, lateral carinae present at basal 0.5. Prepectal carina complete, weak, laterally obscure. Precoxal sulcus deep, coarsely crenulate. Metanotum with median carina. Propodeum coarsely rugose-reticulate, weakly rugose-punctate basolaterally.

Wings. Fore wing, r: 3-SR: SR1=15: 24: 85. 2-SR: 3-SR: r-m=25: 29: 30. 1-M: m-cu=74: 48. 1-CU1: 2-CU1=3: 28. r-m curved below, with remnant vein. Hind wing, marginal cell widened apically, its apical width 3.0 times minimum width of cell below vein R1. 1-M: 1r-m =21: 19. cu-a strongly inclivous.

Legs. Length of hind femur, tibia and basitarsus 6.0, 11.8 and 9.8 times their width, respectively. Hind tibia 1.8 times as long as hind femur.

Metasoma. First tergite rather slender and widened posteriorly, coarsely rugose, but medio-apically smooth, dorsal carinae present in basal half. Length of first tergite 3.3 times its apical width. Second and following tergites smooth.

Colour. Body black. Antenna brown, but 10th-15th flagellomeres whitish yellow. Most of mandible reddish brown, apex of teeth black. Palpi yellow. Fore and middle legs, hind trochanters yellow, hind coxa and femur reddish brown, hind tibia dark brown, hind tarsus whitish yellow. Tegulae and pterostigma dark brown. Wing membrane yellowish brown with veins brown to dark brown.

Female. Unknown.

#### Diagnosis.

This new species is similar to *Brulleia flavibasalis* He and Chen, but differs in having the apical margin of labium convex medially (in latter truncate apically); side of pronotum punctate, antero-medially and subdorsally crenulate, and ventro-posteriorly rugose-punctate (in latter crenulate antero-medially and subdorsally, remainder rather smooth); scutellum densely punctate (in latter rather smooth) and most of the body black (in latter brownish yellow).

#### Distribution.

China (Guizhou).

#### Etymology.

It is named after the type locality of the species, Fanjing Mountain in Guizhou Province of China.

**Figures 1–10.**
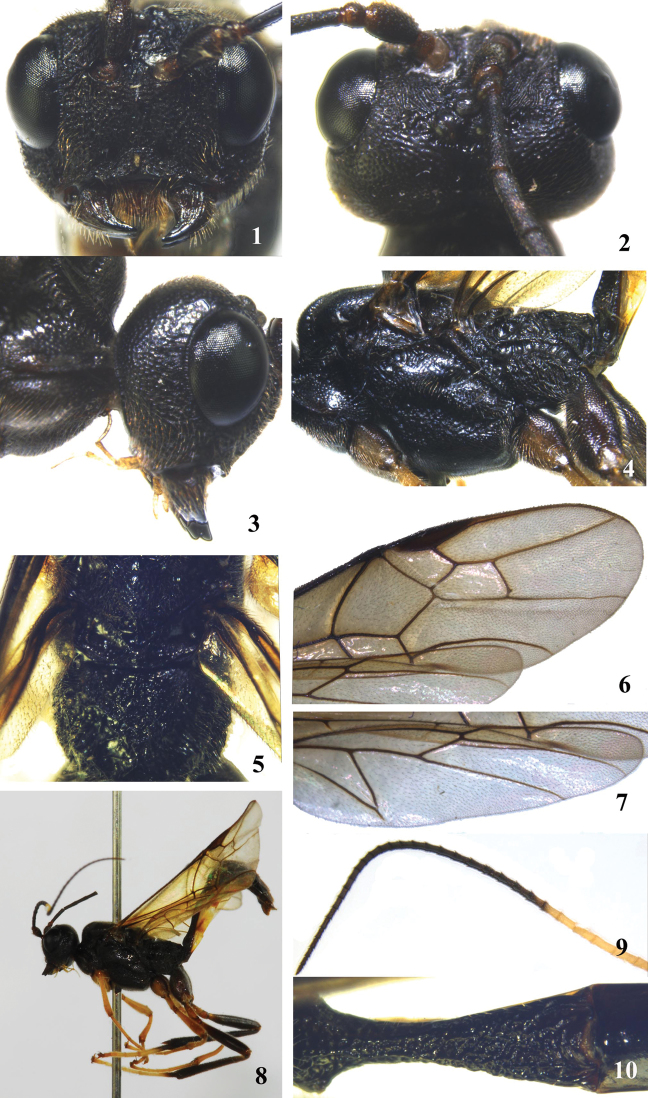
*Brulleia fanjingensis*, sp. n. **1** head, frontal aspect **2** head, dorsal aspect **3** head, lateral aspect **4** mesosoma, lateral aspect **5** propodeum, dorsal aspect; **6** fore wing **7** hind wing **8** habitus, lateral aspect **9** 10th-42th flagellomeres **10** first and basal second metasomal tergites, dorsal aspect.

### Brulleia longipalpis

Yan & Chen, sp. n.

urn:lsid:zoobank.org:act:F1A04DF2-9445-4AF6-A3B4-0FEAD60D57CF

http://species-id.net/wiki/Brulleia_longipalpis

Figs 11–19

#### Material examined.

Holotype, ♀, China, Tibet, Motuo, 1570 m, 21.V.1980, Jin Gentao and Wu Jianyi, No. 34201363 (SEMS).

#### Description.

Body length (excluding ovipositor sheath) 16 mm. Fore wing length 13.5 mm.

Head. Antennal segments 40; third segment 1.2 times longer than fourth segment; length of third, fourth and penultimate segments 5.0, 4.3 and 1.1 times their width, respectively. Maxillary palp 6-segmented; labial palp 3-segmented; length of maxillary palp 1.1 times height of head. Head in dorsal view 0.6 times as long as wide. Eye 1.1 times as long as temple in dorsal view. Length of malar space 0.9 times basal width of mandible, 0.4 times maximum width of eye. POL: OD: OOL=10: 10: 27. Vertex and temple densely punctate. Frons slightly concave, medially punctate with some rugae, obliquely rugose-punctate laterally. Face reticulate-punctate. Clypeus rugose-punctate, its apical margin slightly convex with median notch. Labium concave medially, apically with median notch.

Mesosoma. Length 1.8 times its height. Pronope deep, spindle-shaped. Side of pronotum punctate, antero-medially crenulate, subdorsally rugose-punctate, posteriorly reticulate-punctate. Notauli narrow and deep, crenulate. Scutellar sulcus with one carina and several lateral crenulae. Scutellum densely punctate, lateral carinae absent, with several striae posteriorly. Precoxal sulcus complete and wide, coarsely rugose-punctate. Metanotum with two short carinae. Propodeum coarsely reticulate, finely punctate basolaterally, coarsely rugose postero-laterally.

Wings. Fore wing, r: 3-SR: SR1=12: 20: 72. 2-SR: 3-SR: r-m=17: 20: 19. 1-M: m-cu=39: 30. 1-CU1: 2-CU1=5: 50. r-m curved slightly below, without remnant vein. Hind wing, marginal cell obviously widened apically, its apical width 3.0 times minimum width of cell below vein R1. 1-M: 1r-m=35: 22. cu-a inclivous.

Legs. Length of hind femur, tibia and basitarsus 6.0, 13.3 and 10.0 times their width, respectively. Hind tibia 1.8 times as long as hind femur.

Metasoma. First tergite reticulate-punctate, medio-posteriorly smooth, dorsal carinae distinct in basal 0.3. Length of first tergite twice its apical width. Ovipositor sheath 3.4 times as long as metasoma, 3.7 times as long as hind tibia, 5.0 times as long as mesosoma, and 2.3 times as long as fore wing.

Colour. Body black. Antenna dark brown but 10th-17th flagellomeres yellow. Palpi yellow. Tegulae, basal of mandible and labium reddish brown. Legs yellow to reddish yellow but coxae reddish brown, hind tarsus whitish yellow. Second tergite reddish yellow at two-thirds basolaterally. Ovipositor sheath dark brown. Wing membrane fumose with veins dark brown.

Male. Unknown.

#### Diagnosis.

This new species is similar to *Brulleia obereae* Chen and van Achterberg, but differs in having the maxillary palp longer, its length 1.1 times height of head (in latter 0.5 times); temple densely punctate (in latter sparsely and finely punctulate dorsally, and rugose ventrally) and first tergite mainly reticulate-punctate, but medio-posteriorly smooth (in latter basally transversely, medially irregularly and apically more or less longitudinally rugose).

#### Distribution.

China (Tibet).

#### Etymology.

It is named after its very longmaxillary palp.

**Figures 11–19.**
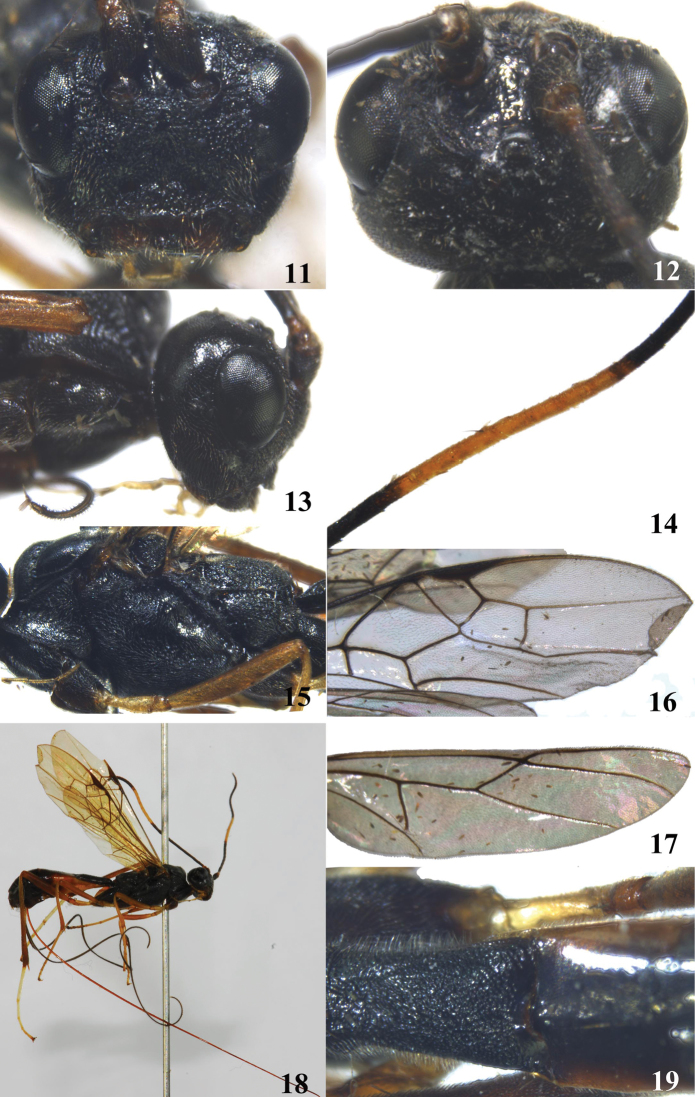
*Brulleia longipalpis*, sp. n. **11** head, frontal aspect **12** head, dorsal aspect **13** head, lateral aspect **14** 10th-17th flagellomeres **15** mesosoma, lateral aspect **16** fore wing **17** hind wing **18** habitus, lateral aspect **19** first and basal second metasomal tergites, dorsal aspect.

### Brulleia noncarinata

Yan &Chen, sp. n.

urn:lsid:zoobank.org:act:1D4A8931-D486-4789-90D8-9F8A348B158F

http://species-id.net/wiki/Brulleia_noncarinata

Figs 20–28

#### Material examined.

Holotype, ♀, China, Tibet, Motuo, 1520 m, 8.VII.1980, Jin Gentao and Wu Jianyi, No. 34202321 (SEMS).

#### Description.

Body length (excluding ovipositor sheath) 18.5 mm. Fore wing length 15.2 mm.

Head. Antennal flagellomeres missing. Maxillary palp 4-segmented; labial palp 3-segmented; length of maxillary palp 0.5 times height of head. Head in dorsal view 0.5 times as long as wide. Eye 1.4 times as long as temple in dorsal view. Length of malar space equal to basal width of mandible, 0.4 times maximum width of eye. POL: OD: OOL = 8: 12: 28. Vertex punctate. Temple punctate dorsally, densely rugose-reticulate ventrally. Frons concave, medially almost smooth with some rugae, laterally with slightly oblique striae. Face densely reticulate-punctate. Clypeus rugose-punctate, its apical margin convex and with median notch, ventrally with obscure transverse striae. Labium punctate, truncate apically, slightly concave medially.

Mesosoma. Length 1.7 times its height. Side of pronotum punctate, antero-medially, postero-medially and dorsally crenulate. Notauli narrow and shallow, crenulate. Mesoscutum densely punctate. Scutellum weakly convex, smooth medially, punctate laterally, with several striae posteriorly. Prepectal carina complete, weak, laterally obscure. Precoxal sulcus complete, anteriorly reticulate, rugose crenulate medially, posteriorly longitudinally punctato-striate, ventrally irregularly reticulate-punctate. Scutellar sulcus with single carina. Metanotum with median carina. Propodeum coarsely rugose-reticulate, almost smooth basolaterally.

Wings. Fore wing, r: 3-SR: SR1=16: 27: 88. 2-SR: 3-SR: r-m=24: 27: 22. 1-M: m-cu=34: 23. 1-CU1: 2- CU1=7: 63. r-m curved slightly below, without remnant vein. Hind wing, marginal cell obviously widened apically, its apical width 2.5 times minimum width of cell below vein R1. 1-M: 1r-m=31: 20. cu-a inclivous.

Legs. Length of hind femur, tibia and basitarsus 5.8, 12.8 and 11 times their width, respectively. Hind tibia 1.7 times as long as hind femur.

Metasoma. First tergite widened posteriorly, densely punctate, postero-laterally longitudinally punctate-striate, postero-medially obscurely punctate, dorsal carinae absent. Length of first tergite 3.0 times its apical width. Second and following tergites smooth and shinny. Ovipositor sheath 1.9 times as long as metasoma, 2.1 times as long as hind tibia, 2.5 times as long as mesosoma, and 1.1 times as long as fore wing.

Colour. Body black. Malar space apically, base of mandible and labium dark red. Palps yellowish brown. Tegulae dark brown. Coxae, hind femur and apical one-fourth of hind tibia dark reddish brown; trochanters and tarsus whitish yellow; fore and middle femora, tibiae and basal three-fourthes of hind tibia yellowish brown. First-third metasomal sternites yellowish brown. Pterostigma and most of veins dark brown, wing membrane fumose.

Male. Unknown.

#### Diagnosis.

This new species is similar to *Brulleia flavibasalis* He and Chen, but differs in having the clypeus rugose-punctate, ventrally with obscure transverse striae, its apical margin convex and with median notch (in latter finely rugose, its apical margin slightly concave and without median notch); the dorsal carinae of first tergite absent (in latter present in basal half) and the most part of the body black (in latter brownish yellow).

#### Distribution.

China (Tibet).

#### Etymology.

From ““*non*” (Latin for “absent”), and “*carina*” (Latin for “carina”), because dorsal carinae of the first tergite absent.

**Figures 20–28.**
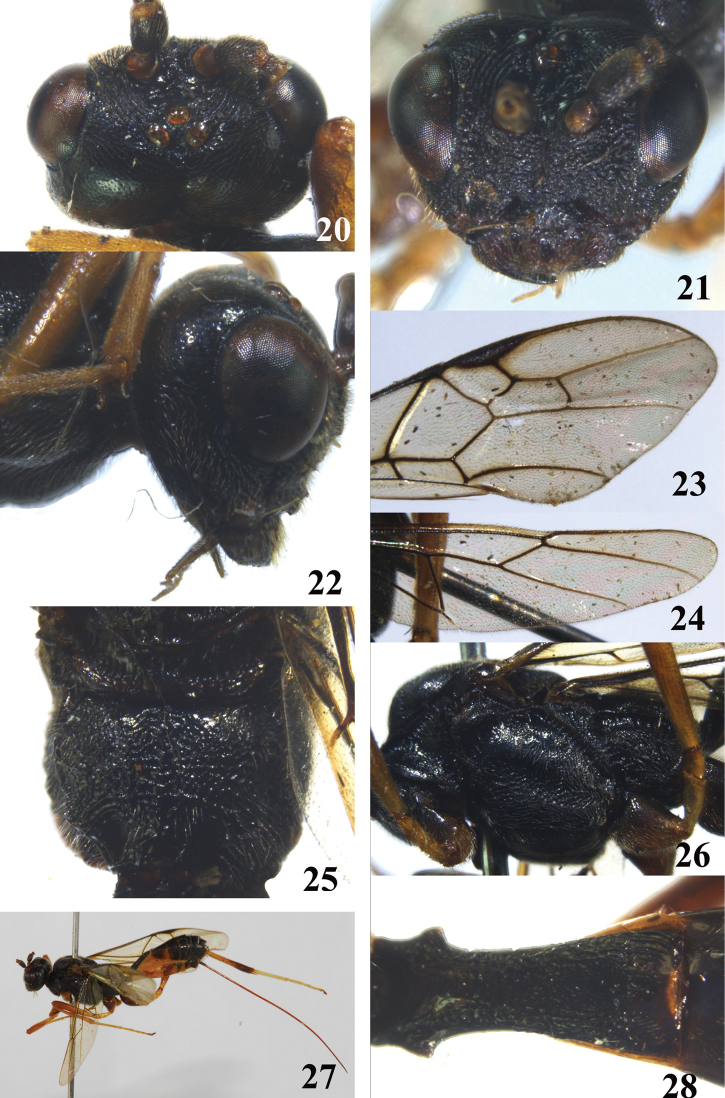
*Brulleianoncarinata*, sp. n. **20** head, dorsal aspect **21** head, frontal aspect **22** head, lateral aspect **23** fore wing **24** hind wing **25** propodeum, dorsal aspect **26** mesosoma, lateral aspect **27 **habitus, lateral aspect **28** first metasomal tergite, dorsal aspect.

### Brulleia punctata

Yan &Chen, sp. n.

urn:lsid:zoobank.org:act:0582609A-F976-4115-AC2C-8A8A88C9F5E1

http://species-id.net/wiki/Brulleia_punctata

Figs 29 37

#### Material examined.

Holotype, 1♀, China, Hebei, Chahar, Yangkiaping, 21.VII.1937, O. Piel, No. 201105603 (ZJUH). Paratype: 1♀, China, Hebei, Chahar, Yangkiaping, 21.VII.1937, O. Piel, No. 201105604 (ZJUH).

#### Description.

Body length (excluding ovipositor sheath) 16.5 mm. Fore wing length 12.2 mm.

Head. Antennal segments more than 33 (apical segments missing); third segment 1.3 times longer than fourth segment; length of third and fourth segments 4.0 and 3.0 times their width, respectively. Maxillary palp 4-segmented; labial palp 3-segmented; length of maxillary palp 0.7 times height of head. Head in dorsal view 0.6 times as long as wide. Eye 1.3 times as long as temple in dorsal view. Length of malar space equal to basal width of mandible, 0.3 times maximum width of eye. POL: OD: OOL=9: 10: 24. Vertex densely punctate. Temple punctate dorsally, densely reticulate-punctate ventrally. Frons slightly concave, medially almost smooth with some rugae, laterally with oblique striae. Face densely rugose-reticulate, medially with a triangular promience near antennal sockets. Clypeus reticulate-punctate, apical margin with median notch, ventrally with transverse striae.

Mesosoma. Length 1.6 times its height. Pronope deep, slit-shaped. Side of pronotum punctate, antero-medially, subdorsally and posteriorly crenulate, postero-medially almost smooth. Notauli narrow and deep, crenulate, its posterior area with median carina. Scutellum slightly convex and densely punctate, lateral carinae absent, with several striae posteriorly. Prepectal carina complete, weak, laterally obscure. Precoxal sulcus coarsely crenulate, anteriorly rugose-punctate. Metanotum without median carina. Propodeum coarsely rugose-reticulate.

Wings. Fore wing, r: 3-SR: SR1=17: 20: 60. 2-SR: 3-SR: r-m=15: 20: 21. 1-M: m-cu=40: 25. 1-CU1: 2-CU1=10: 51. r-m slightly curved below, without remnant vein. Hind wing, marginal cell widened apically, its apical width 2.5 times minimum width of cell below vein R1. 1-M: 1r-m=20: 14. cu-a inclivous, posteriorly slightly curved towards wing base.

Legs. Length of hind femur, tibia and basitarsus 6.4, 13.7 and 10.7 times their width, respectively. Hind tibia 1.8 times as long as hind femur.

Metasoma. First tergite robust and widened posteriorly, densely rugose, smooth apically, dorsal carinae obscure. Length of first tergite 1.8 times its apical width. Second and following tergites smooth and shinny. Ovipositor sheath twice as long as metasoma, 2.5 times as long as hind tibia, 2.7 times as long as mesosoma, and 1.3 times as long as fore wing.

Colour. Body dark reddish brown. Face, clypeus and mandible yellowish brown. Antennal scapus and pedicel yellowish brown, 8th-14th flagellomeres and palpi yellow. Tegula, metasoma (except first tergite) and legs reddish-brown. Ovipositor sheath dark brown. Pterostigma yellowish brown, veins dark brown to light brown, wing membrane faintly brown.

#### Variation.

Body length (excluding ovipositor sheath) 12.8-16.5 mm, fore wing length 10.3–12.2 mm. First tergite dorsal carinae obscure or only visible basally.

Male. Unknown.

#### Diagnosis.

This new species is similar to *Brulleia flavibasalis* He and Chen, but differs in having the frons medially almost smooth with some rugae, laterally with oblique striae (in latter concave medially, with sparse, fine and obsolete punctures laterally); the length of maxillary palp 0.7 times height of head (in latter 0.5 times) and dorsal carinae of first tergite obscure or only visible basally (in latter present in basal half).

#### Remark.

The tarsus of left hindleg of holotype missing. Most of the flagellomeres of paratype missing.

#### Distribution.

China (Hebei).

#### Etymology.

After dense punctation of scutellum.

**Figures 29–33.**
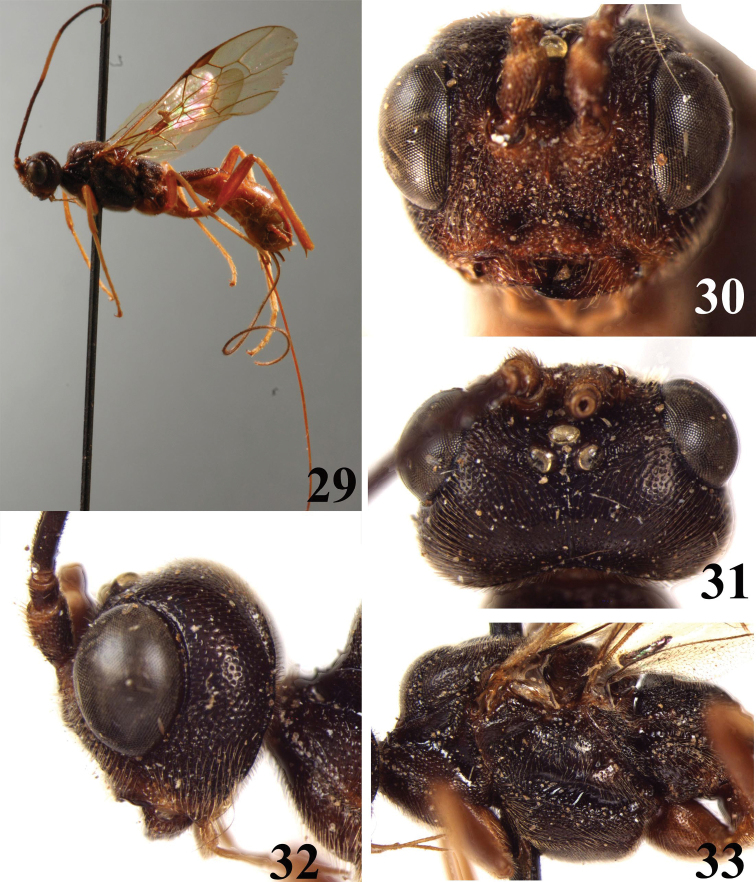
*Brulleia punctata*, sp. n. **29** habitus, lateral aspect **30** head, frontal aspect **31** head, dorsal aspect **32** head, lateral aspect **33** mesosoma, lateral aspect.

**Figures 34–37.**
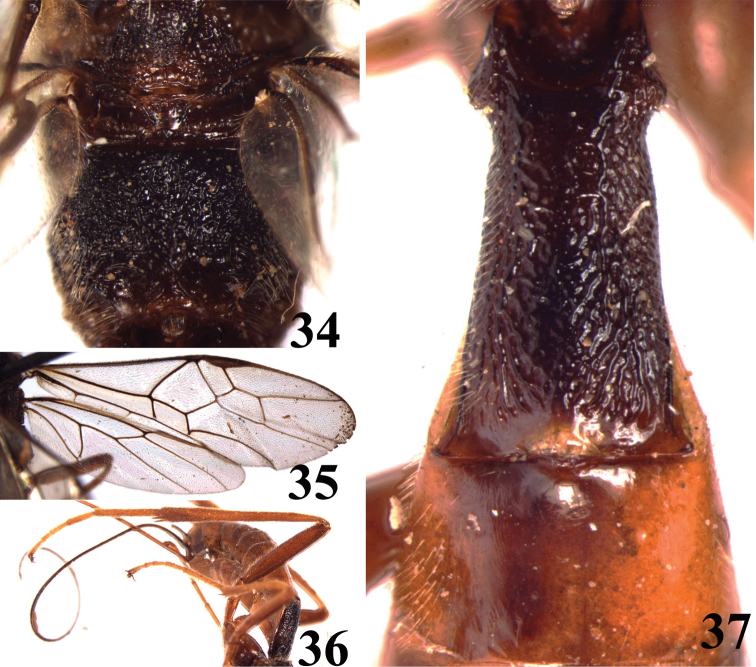
*Brulleia punctata*, sp. n. **34** propodeum, dorsal aspect **35** fore and hind wings **36** Hind leg, lateral aspect **37** first and second metasomal tergites, dorsal aspect.

##### Key to the Chinese species of the genus *Brulleia* Szépligeti

**Table:** 

1	Maxillary palp with 4 segments	2
–	Maxillary palp with 5-6 segments	5
2	Scutellum densely punctate	3
–	Scutellum almost smooth, at most with some punctation laterally	4
3	Middle mesoscutal lobe with weak longitudinal groove medially; scutellum rather flat and lateral carinae present at basal 0.5; metanotum with median carina; first tergite rather slender; length of first tergite 3.3 times its apical width; metasoma black. Guizhou	*Brulleia fanjingensis* sp. n.
–	Middle mesoscutal lobe normal, without longitudinal groove medially; scutellum slightly convex and lateral carinae absent; metanotum without median carina; first tergite robust; length of first tergite 1.8 times its apical width; metasoma (except first tergite) reddish brown. Hebei	*Brulleia punctata* sp. n.
4	Body brownish yellow; first tergite densely rugose, postero-medially polished, dorsal carinae present in basal half; clypeus finely rugose, its apical margin slightly concave and without median notch. Guangxi	*Brulleia flavibasalis* He & Chen
–	Body black; first tergite densely punctate, postero-laterally longitudinally punctato-striate, postero-medially obscurely punctate, dorsal carinae absent; clypeus rugose-punctate, ventrally with obscure transverse striae, its apical margin convex and with median notch. Tibet	*Brulleia noncarinata* sp. n.
5	Body yellowish brown to reddish brown; vein 1-M of hind wing 0.8–1.5 times vein 1r-m; vein cu-a of hind wing comparatively less inclivous	6
–	Body black, only second tergite and its surrounding area dark reddish (or reddish-yellow basally); vein 1-M of hind wing 1.5–2.2 times vein 1r-m; vein cu-a of hind wing strongly inclivous	8
6	Antenna black with yellowish white submedian band; pterostigma reddish yellow to yellowish brown. Fujian, Zhejiang	*Brulleia rubida* Chen & He
–	Basal half of antenna reddish yellow or brownish yellow, apical half black; color of pterostigma variable	7
7	First tergite densely rugose, transversely medially, and its dorsal carinae present extremely basally; second tergite rugulose basolaterally; temple smooth dorsally, with coarse punctures ventrally; wing membrane dark yellowish brown; vein 3–SR slightly longer than veins 2-SR or r-m; length of hind femur 5.8 times its width. Guangxi	*Brulleia lutea* He & Chen
–	First tergite smooth basally and apically, its basal 0.2-0.5 transversely rugose, remaining part irregularly rugose, dorsal carinae present at most of basal half; second tergite polished; temple punctulate dorsally, rugose-punctate ventrally; wing membrane yellowish brown; vein 3–SR slightly shorter than veins 2-SR or r-m; length of hind femur 8.6 times its width. Sichuan	*Brulleia yangi* He & Chen
8	Second tergite distinctly rugose medially, only apically and laterally smooth; length of first tergite 3.1–3.4 (♂) times its apical width	9
–	Second tergite almost smooth, at most obscurely rugose basally or rugose basolaterally; length of first tergite 1.9–2.9 (♂) and 2.1–2.6 (♀) times its apical width	10
9	Length of maxillary palp 0.55 times height of head; vein cu-a of fore wing almost interstitial; apical width of marginal cell of hind wing about 1.8 times minimum width of cell below vein R1; vein 1-M of hind wing about 1.5 times vein 1r-m. Guizhou	*Brulleia tenuipetiolata* Chen & He
–	Length of maxillary palp 0.7 times height of head; vein cu-a of fore wing obviously postfurcal; apical width of marginal cell of hind wing about 2.3 times minimum width of cell below vein R1; vein 1-M of hind wing about 2.2 times 1r-m. Yunnan	*Brulleia chaoi* Chen & He
10	Second tergite rugose basolaterally, remainder smooth; fourth segment of maxillary palp 1.8 times longer than fifth segment. Fujian	*Brulleia auripes* Chen & He
–	Second tergite smooth, at most obscurely rugulose basally	11
11	Propodeum punctate basolaterally, subbasally finely rugulose; 2-SR: 3-SR: r-m=7: 7: 7. - Length of maxillary palp about 0.7 times height of head. Sichuan	*Brulleia subtilirugula* He & van Achterberg
–	Propodeum coarsely reticulate except its baso-lateral punctated area; 2-SR: 3-SR: r-m=7: 7–9: 7.7–8.5	12
12	Ovipositor sheath 2.3 times as long as fore wing; length of first tergite 2.0 (♀) times its apical width; propodeum coarsely reticulate, finely punctate basolaterally, coarsely rugose postero-laterally. Tibet	*Brulleia longipalpis* sp. n.
–	Ovipositor sheath 1.4–1.5 times as long as fore wing; length of first tergite 2.1-2.6 (♀) times its apical width; propodeum coarsely reticulate or rugose-reticulate except its baso-lateral punctate area	13
13	Frons smooth, medially with two submedial carinae, laterally with oblique striae; clypeus finely rugose; length of maxillary palp about 0.5 times height of head. Jiangxi	*Brulleia obereae* Chen & van Achterberg
–	Frons rugose; clypeus reticulate-rugose, with small rather smooth triangular area medio-ventrallly; length of maxillary palp about 1.1 times height of head. Taiwan	*Brulleia taiwanensis* Chou & Hsu

## Supplementary Material

XML Treatment for Brulleia

XML Treatment for Brulleia fanjingensis

XML Treatment for Brulleia longipalpis

XML Treatment for Brulleia noncarinata

XML Treatment for Brulleia punctata

## References

[B1] van Achterberg C (1983) A revision of the new tribe Brulleiini (Hymenoptera: Braconidae). Contributions of the American Entomological Institute 20: 281-306

[B2] van Achterberg C (1988) Revision of the subfamily Blacinae Foerster (Hymenoptera, Braconidae). Zoologische Verhandelingen (Leiden) 249: 1-324

[B3] van Achterberg C (1993) Illustrated key to the subfamilies of the Braconidae (Hymenoptera: Ichneumonoidea). Zoologische Verhandelingen (Leiden) 283: 1-189

[B4] Belokobylskij SA (1998) Subfam. Helconinae. In: Lehr PA (Ed.). Opredelitel’ nasekomykh Dal’nego Vostoka Rossii.Tom 4. Setchato krylo obraznye, skorpionnitzy, pereponchatokrylye. Chast’ 3. Dal’nauka, Vladivostok: 411-435 [in Russian]

[B5] Chen XX He JH van Achterberg C (1993) A revision of the subtribe Brulleiina van Achterberg (Hymenoptera: Braconidae: Helconinae) from China. Zoologische Mededelingen (Leiden) 67(27–43)): 375-395

[B6] Chen XX He JH Ma Y (1998) Hymenoptera: Braconidae (I). In: Wu H (Ed). Insects of Longwangshan Nature Reserve.‘ China Forestry Publishing House, Beijing: 392-394 [in Chinese with English summary]

[B7] Chen XX He JH Ma Y (2001) Hymenoptera: Braconidae. In: Wu H Pan CW (Eds). Insects of Tianmushan National Nature Reserve.Science Press, Beijing: 723-733 [in Chinese with English summary]

[B8] Chou LY Hsu TC (1998) The Braconidae (Hymenoptera) of Taiwan 8. Brulleiini, Diospilini and Helconini. Journal of Agricultural Research of China 47 (3): 283-314

[B9] Szépligeti G (1904) Hymenoptera. Fam. Braconidae. Genera Insectorum, 22: 1-253

[B10] Shenefelt RD (1970) Braconidae 2. Helconinae, Calyptinae, Mimagathidinae, Triaspinae. Hymenopterorum Catalogus (nova editio). pars 5, 177–306

